# Multi-functional self-assembly nanoparticles originating from small molecule natural product for oral insulin delivery through modulating tight junctions

**DOI:** 10.1186/s12951-022-01260-9

**Published:** 2022-03-05

**Authors:** Xiaohui Jia, Zhihua Yuan, Yuqin Yang, Xuemei Huang, Nana Han, Xiaojing Liu, Xiaoyu Lin, Tao Ma, Bing Xu, Penglong Wang, Haimin Lei

**Affiliations:** School of Chinese Pharmacy, Beijing University of Chinese Medicine, Beijing, 102488 People’s Republic of China

**Keywords:** Oral insulin, Small molecule natural product, Multi-functional nanoparticles, pH-sensitive, Mucoadhesive, Tight junction

## Abstract

**Background:**

Oral administration of insulin (INS) could be absorbed into systemic circulation only if the carrier protected it from the hostile gastrointestinal conditions. However, traditional macromolecular carriers have not totally overcome challenges in addressing these biological barriers.

**Result:**

In this study, inspired by small molecule natural products (SMNPs), we demonstrate the multi-functional self-assembly nanoparticles (BA-Al NPs) originating from baicalin (BA) and AlCl_3_ through coordination bonds and hydrogen bonds. As a novel carrier for oral insulin delivery (INS@BA-Al NPs), it displayed effective capacity in pH stimuli-responsive insulin release, intestinal mucoadhesion and transepithelial absorption enhance. Meanwhile, BA improved the paracellular permeability for insulin absorption, because of its downregulation at both mRNA and protein level on internal tight junction proteins. In vivo experiments exhibited remarkable bioavailability of INS and an ideal glucose homeostasis in the type I diabetic rat model.

**Conclusion:**

This study offers a novel frontier of multi-functional carriers based on SMNPs with self-assembly character and bioactivity, which could be a promising strategy for diabetes therapy.

**Graphical Abstract:**

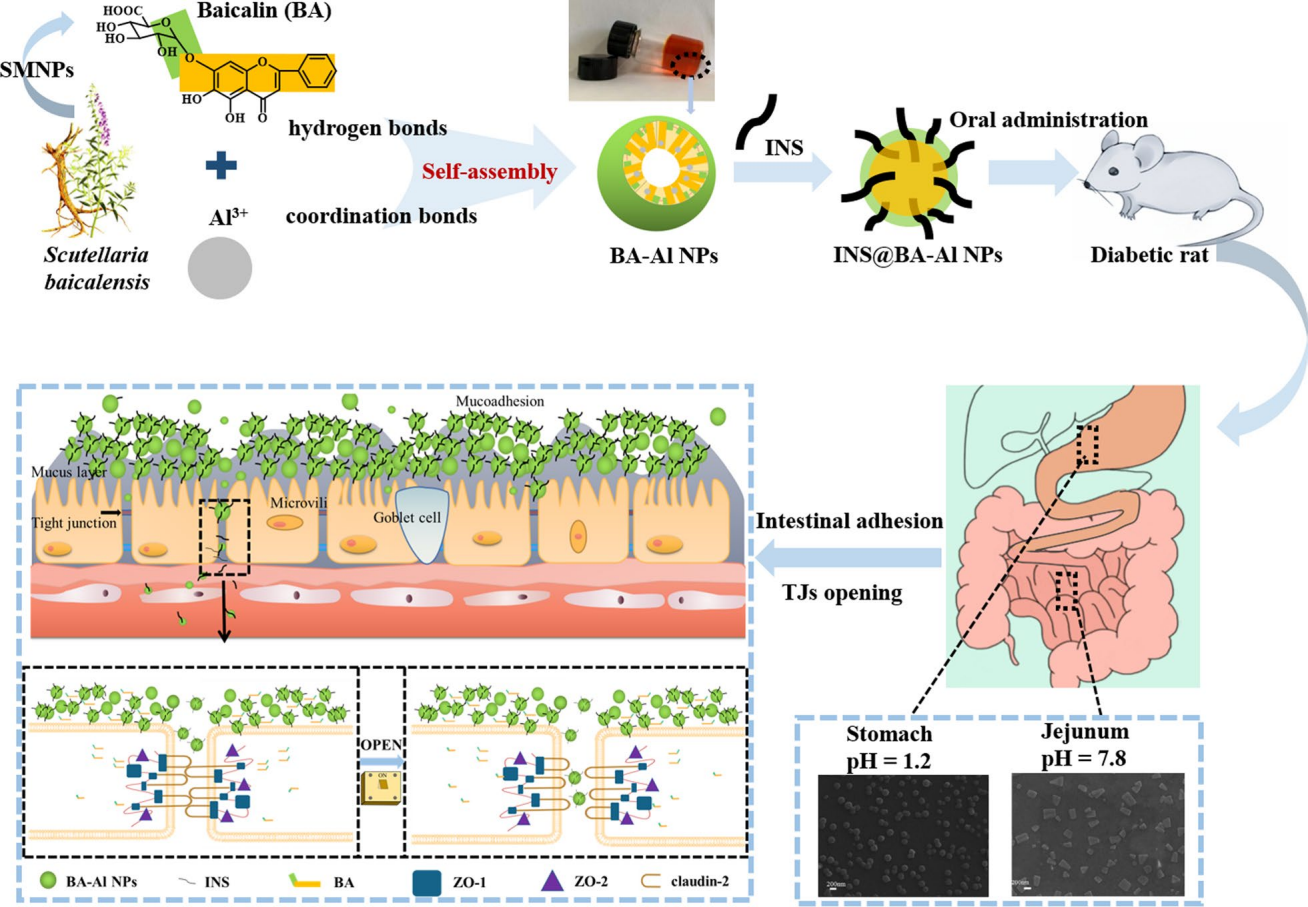

**Supplementary Information:**

The online version contains supplementary material available at 10.1186/s12951-022-01260-9.

## Introduction

Diabetes mellitus is a pervasive, insidious and lifelong metabolic disease that threatens the health of 466 million people in the world in 2021 according to a report from the World Health Organization and this number is anticipated to increase to 625 million by 2040 worldwide [1, 2]. To date, subcutaneous parenteral still remain the conventional way to deliver insulin (INS) daily. However, subcutaneous injections of INS treatment for type 1 diabetes always causes patients pain and local tissue infection [3, 4]. Orally administered INS, on the other hand, has received a lot of attention due to its patient friendly, non-invasive and convenient character [5–7]. Unfortunately, the bioavailability of oral INS is still limited (less than 2%) by harsh biological barriers including gastric acid and intestinal mucosal [8, 9]. The strategy to overcome these barriers is to use functional platforms, which protects INS from violent pH fluctuations in gastrointestinal tract, at the same time that improve the permeability via paracellular or transcellular transport and safety. [10, 11]. The existing carriers produced by natural/synthetic macromolecule (such as chitosan [12], hyaluronic acid [13], etc*.*) are used to meet the requirement [14, 15]. However, these carriers need conjugate ligands such as cell-penetrating peptides [16], surfactants and *zonula occludens* toxin to open the tight junctions (TJs) in paracellular way [17], which may damage the intestinal epithelium[18]. In addition, they require synthesis or structural modification, which might cause toxicity and side effects during degradation and metabolism in the human body [19]. The deficiencies of carriers based on macromolecule might have hindered the clinical translation of oral INS. Therefore, it is urgent to search for a safe and multi-functional carrier for oral INS delivery.

Small molecule natural products (SMNPs) have been extensively investigated owing to their available pharmacological activity, good biocompatibility and long history of clinical usage [20, 21]. With the continuous development of supramolecular self-assembly technology, their application as a part of biomedically functional delivery vehicles has been proposed to construct in drug delivery system without any structural modification or organic solvent [22, 23]. The delivery carriers produced by specific SMNPs not only have satisfactory safety but also have stimuli-sensitive characteristics to control the release of agents [24]. More importantly, SMNPs confer significant bioactive activities, which can further play a synergistic role to treat disease without the help of any additives [25]. Therefore, compared with large molecular compounds, the carrier prepared by self-assembly technology of SMNPs have many significant advantages in oral INS transport. However, to our best knowledge, these carriers have not been exploited so far, which has greatly hindered the rapid development in self-assembled SMNPs and oral INS field.

**Fig. 1:**
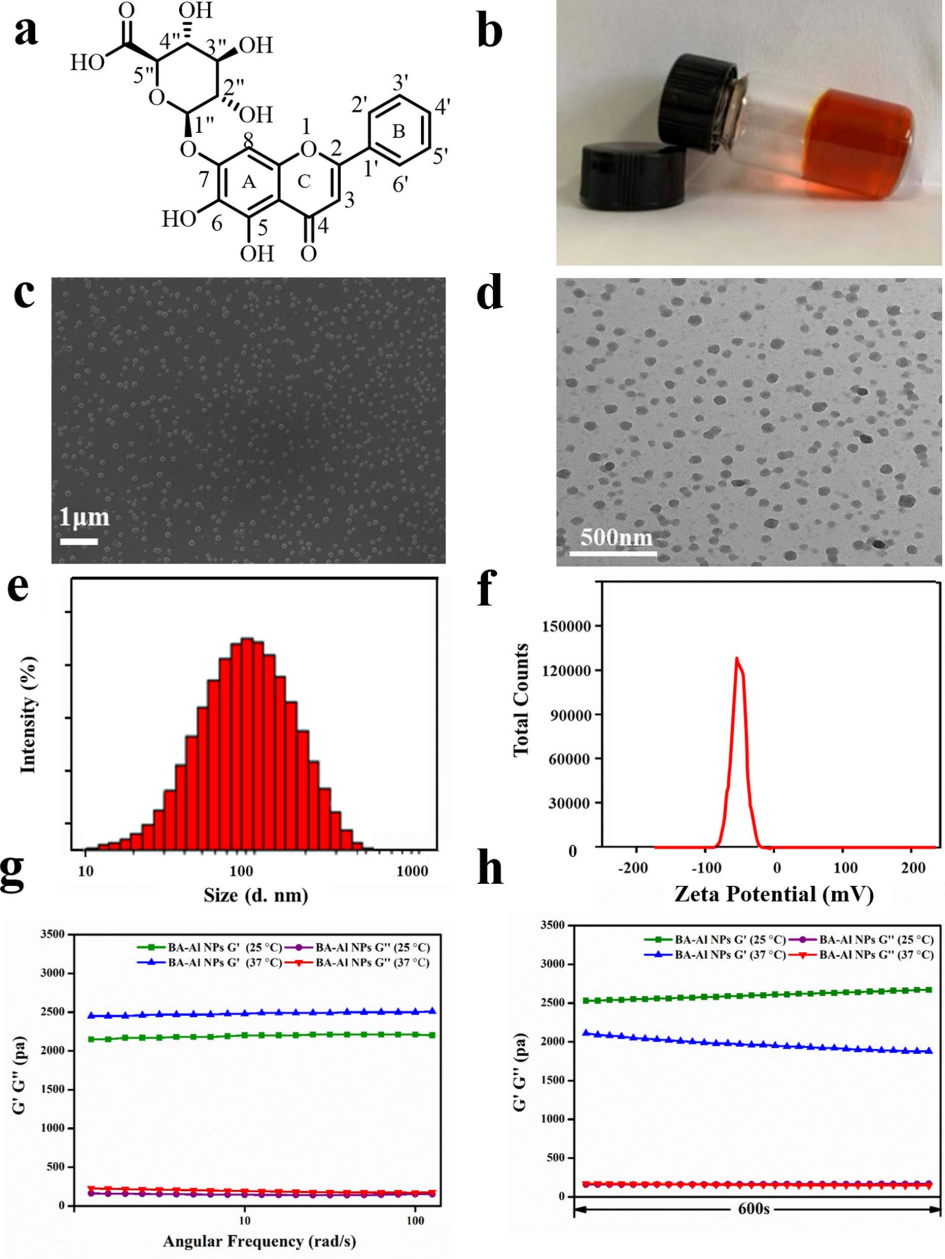
Chemical structure of BA (**a**); Macrograph of the hydrogel (**b**); FESEM image of the nanoparticles (**c**); TEM image of the nanoparticles (**d**); The hydrodynamic diameters of the nanoparticles (**e**); The zeta potential of the nanoparticles (**f**); The storage modulus (G′) and loss modulus (G″) of the hydrogel as a function of angular frequency at 25 °C and 37 °C (**g**); The storage modulus (G′) and loss modulus (G″) of hydrogel at 25 °C and 37 °C in 600 s (**h**)

Baicalin (BA, Fig. 1a), as a SMNPs, belonging to flavonoids family separated from *Scutellaria baicalensis*, has been broadly used for different applications in the research and clinic. Our previous study has found that BA has self-assembled character to form nanoparticles with enhancement effect and good biocompatibility [26]. Recently, it has been reported that BA could act as an effective absorption enhancer to modulate integrity of the TJs, which may benefit to the INS oral administration [27]. Despite all the advantages of the BA, there is no multi-functional carrier that can combine these advantages to achieve the application of oral INS.

In this work, BA and AlCl_3_ were used to prepare nanoparticles (BA-Al NPs) via facile and green self-assembly strategy without any organic solvent and complicated preparation process to delivery INS for oral administration (INS@BA-Al NPs). Combing with a series of spectral, mass spectrometry and thermodynamic methods, we found that BA-Al NPs were mainly driven by hydrogen bonds and metal–ligand coordination bonds. Serving as a protein delivery vehicle and drug permeability enhancer, BA-Al NPs could protect INS from being degraded under severe acidic conditions in stomach, elicit release under alkaline condition in intestine and extend retention time. In addition, BA-Al NPs disassembled and released BA to downregulate the TJs proteins expression and further improved the absorption of INS to maintain glucose homeostasis through modulating TJs. Meanwhile, in vivo experiments exhibited remarkable bioavailability (8.88 ± 1.05%) of INS and an ideal glucose homeostasis in the diabetes model. The combination of multi-functional biomaterial and significant therapeutic performance makes the naturally derived BA-Al NPs nanocarrier a promising platform for drug delivery system.

## Materials and methods

### Preparation of the BA-Al NPs and INS@BA-Al NPs

BA-Al NPs were prepared via one-pot preparation procedures. Briefly, BA was dispersed in deionized water and adjusted to pH = 7 with sodium hydroxide (NaOH) solution under vigorous stirring to obtain the BA solution. Afterward, AlCl_3_ was added into BA solution at 60 °C under a constant BA / AlCl_3_ molar ratio of 2:1. After stirring for 2 h, the mixture of BA and AlCl_3_ formed the self-assembled BA-Al NPs. For INS-loaded BA-Al NPs preparation, INS was firstly dissolved in hydrochloric acid solution (pH = 2) before being added into BA-Al NPs. After stirring overnight, INS-loaded BA-Al NPs (INS@BA-Al NPs) was obtained and stored at 4 °C for future use. During the encapsulation of BA-Al NPs, maintaining the structural integrity of INS under different pH conditions is crucial to its therapeutic effect. Ultraviolet-visible circular dichroism (Far-UV CD) spectrophotometry and fluorescence spectra were used to monitor the secondary structure of INS under different pH conditions (Additional file 1: Fig. S1).

### Characterization of the BA-Al NPs

The morphology of BA-Al NPs was imaged on scanning electron microscopy (SEM, ZEISS-SUPRA55, Germany) and transmission electron microscopy (TEM, JEM 2100F, JOEL, Tokyo, Japan). Dynamic light scattering (DLS) and zeta potential of the nanoparticles were carried out to collect the size distribution and charge properties using a Malvern Zetasizer Nano-ZS (DLS; Zetasizer Nano ZS 90, Malvern Instrument, UK). Loss modulus (G'') and storage modulus (G') measurements were operated on a rotated rheometer (Physica MCR301, Aaton paar, Austria). To explore the effects of different temperatures on nanoparticles, the results were measured at 25 ℃ and 37 ℃. In parallel-plate mode, the dynamic frequency scanning was performed at 0.5% strain, and the angular frequency was performed at 0.1–100 rds·s^−1^. The scanning time was held for 1340 s at 25 ℃ and 661 s at 37 ℃. An UV–vis spectrophotometer (HITACHI UH5300, Japan) with the scanning range from 200 to 600 nm was used to detect UV spectra of the BA-Al NPs and BA. The basic unit of BA-Al NPs was analyzed by a high-resolution mass spectroscopy (static spray-HRMS, Waters, USA). CD spectra of BA and BA-Al NPs were measured using Chirascan V100 (Applied Photophysics, UK) for the range 200–600 nm. The wavelength and bandwidth were set to 280 nm and 2 nm, respectively. Fourier transform infrared (FT-IR) characterization of BA, and the self-assembled BA-Al NPs was recorded on a Fourier transform infrared spectrometer (Alpha II, Bruker, Germany) with a frequency range of 400–4000 cm^−1^. X-ray diffraction (XRD, D8A25, Bruker, Germany) was carried out on Ultima IV (Rigaku Japan) to identify structure of the BA-Al NPs. The 2-theta was measured at 5°-50° with 40 kW and 40 mA, and the scanning speed was 3° min^−1^.

### In vitro release of insulin from INS@BA-Al NPs

To evaluate the release of the INS@BA-Al NPs, it was dispersed in 5 mL simulated gastric fluid (pH = 1.2) and simulated intestinal fluid (pH = 7.8), respectively. Then it was incubated at 37 ℃ with continuous shaking at 80 rpm using a constant-temperature shaker (SHA-B, Guohua Co., Ltd., China). At specific time intervals, the sample was taken out, analyzed with enzyme linked immunosorbent assay (ELISA, SEKR-0033, Beijing Solarbio Science & Technology Co., Ltd) and the micrographs were recorded.

### Cellular uptake analysis

MDCK cells at logarithmic growth stage were digested by trypsin and inoculated on 35 mm laser confocal dishes with a density of 1 × 10^5^ cells per well. After the cells were adherent to the wall for 24 h, the supernatant was discarded, and the culture medium containing 10 μg/mL free-form Cy7 dye and 10 μg/mL Cy7@BA-Al NPs was added and incubated for 1 h and 4 h respectively. Then the upper culture medium was removed. The cells were gently washed with cold PBS (pH = 7.4) for 3 times and fixed in a 4 ℃ refrigerator with 70% ethanol solution for 30 min. Then the cells were stained with 10 μg/mL DiR and the fluorescence state of the cells was observed under a confocal laser scanning microscopy (CLSM).

### Animal

Male Wistar rats (250 ± 5 g) were provided by the Institutional Animal Care and Use Committee (IACUC) of Beijing University of Chinese Medicine (Beijing, China). The rats received a balanced diet and tap water ad libitum. All experiments were carried out in accordance with the guidelines for the Care and Use of Experiment Animals in IACUC. Diabetes was induced in Wistar rats by single intraperitoneal injection streptozotocin (STZ) in citrate buffer (dissolved in 10 mM citrate buffer at pH = 4.5 at a dose of 75 mg/kg body weight for 3 days to get type I diabetic rats. Two weeks after the STZ treatment, rats were type I diabetic rats when their fasting blood glucose levels exceeded 300 mg/dL.

### Biodistribution and mucoadhesion study of BA-Al NPs

The mucoadhesion and biodistribution of BA-Al NPs were studied via small animal imaging system in vivo (Maestro 2, CRI, USA). Overnight-fasted mice were given two formulations (Free form Cy7, Cy7@BA-Al NPs) by intragastric administration with a Cy7 dosage of 2.5 mg/kg. After 0.5 h, 2 h, 4 h and 12 h, they were anesthetized with 1.5% isoflurane in 100% oxygen. Fluorescence images of whole body of mice were obtained. And then, the animals were opened by a midline incision when they sacrificed. Major organs of mice’s body were removed to observe Cy7 level, including intestine, heart, liver, spleen, lung, and kidneys at different times, and the total amount of fluorescence in the organs was measured by fluorescence spectrometer (IVIS, PerkinElmer, USA).

### Real-time (RT)-PCR analysis

Total RNA was extracted from Caco-2 cells using TRIzol (Takara). In total, 1 μg of the total RNA of each sample was reversely transcribed into cDNA using Prime- Script RT master mix (Takara) in a total volume of 10 μL. cDNA templates were then amplified with specific primers for target genes in the ABI ViiA 7 real-time PCR system (Applied Biosystems) using 2X SYBR Green PCR Master Mix (Applied Biosystems). Expression of gene of each sample was normalized to the endogenous control *β*-actin, and presented as 2^−ΔΔCt^ using the comparative Ct method. Primer sequences for qPCR analyses are listed in Table 1.

**Table 1:** List of primers used in the study

Target	Sense (5’-3’)	Antisense (5’-3’)
ZO-1	CAAGATAGTTTGGCAGCAAGAGATG	ATCAGGGACATTCAATAGCGTAGC
ZO-2	CGGATTCCAGACAAGGT	CCTTCAGAGACCCAGA
Claudin-1	CCAACGCGGGGCTGCAGCT	TTGTTTTTCGGGGACAGGA
*β-actin*	AACTACCTTCAACTCCATCA	GAGCAATGATCTTGATCTTCA

### Western blotting

Total protein was either extracted from Caco-2 cells using ice-cold RIPA buffer (25 mM Tris–HCl, 150 mM NaCl, 1% NP-40, 1% sodium deoxycholate, 0.1% SDS, complete protease cocktail (Roche)). Protein samples at 10 μg were separated by 7.5–15% SDS-PAGE and electrophoretically transferred to polyvinylidene difluoride membranes (Bio-rad). After blocking with 5% non-fat milk, membranes were incubated with primary antibodies overnight at 4 °C. After rinsing, membranes were incubated with appropriate secondary antibody con- jugated with HRP at room temperature for 1 h. The positive immunoreactions were detected with x-ray film (Fuji) by chemiluminescence using an ECL kit (GE Healthcare). The relative expression of proteins was quantified using Image J software (Wayne Rasband, NIH, USA). The antibodies are listed in Table 2.

**Table 2:** List of antibodies used in the study

Antigen	WB dilutions	Source/Host	Company	Catalog number
ZO-1	1:1000	Rabbit	Cell Signaling Technology	#13663
ZO-2	1:1000	Rabbit	Cell Signaling Technology	#2847
Claudin-1	1:1000	Rabbit	Cell Signaling Technology	#13995
*β-actin*	1:1000	Rabbit	Cell Signaling Technology	#4970
Rabbit IgG secondary antibody	1:2000	Goat anti-rabbit IgG-HRP	Cell Signaling Technology	#7074

### Immunofluorescence analysis

Caco-2 cells were rinsed with prewarmed PBS and permeabilized with cold ethanol (4 °C) for 30 min. Nonspecific binding sites were blocked with 1% BSA in PBS for 10 min. Cells were incubated in 1% BSA in PBS with primary antibodies: anti-ZO-1 (20 μg/ml) for 1 h at room temperature. Cells were washed three times with 1% BSA in PBS and incubated with FITC-conjugated goat-anti rabbit antibodies (1:50–100) as appropriate for 1 h at room temperature. Cells were washed three times with 1% BSA in PBS and analysis on a Confocal Laser Scanning Microscope (Leica TCS SP8).

### In vivo pharmacological activity of INS@BA-Al NPs

Type I diabetic rats were fasted overnight and remained free access to water before testing. Model rats were given normal saline (NS) by intragastric administration. BA-Al NPs loaded with INS (INS@BA-Al INS 50 IU/kg) was administered orally to the diabetic animals. Free-form INS (50 IU/kg) was administered orally by intragastric administration as a negative control. Those subjected to subcutaneous (SC) injection of INS solution (5.0 IU/kg) were used as a positive control (n = 5 for each test group). The blood samples were taken from tail vein and glucose level was checked using Bayer’s glucose meter at regular time interval (2 h). Blood samples were collected from the tail vein of rats prior to drug administration and at different time intervals after dosing. The blood glucose levels then measured using a glucose meter (LifeScan Inc., Milpitas, CA, USA).

To evaluate the bioavailability of BA-Al NPs after peroral treatment, the plasma INS level was measured. The experimental method of blood collection is the same as before, and the serum was separated by centrifugation at 5000 rpm for 10 min at 4 ℃, and stored at − 20 ℃. Serum INS concentrations were measured using enzyme-linked immunosorbent assay (ELISA, SEKR-0033, Beijing Solarbio Science & Technology Co.,Ltd). The relative bioavailability (BAR) of test nanoparticles after peroral treatment was calculated using Eq. (1):

1 $${\text{BAR }} = \, \left[ {{\text{AUC}}_{{({\text{oral}})}} \times {\text{ DOSE}}_{{({\text{sc}})}} } \right] \, / \, \left[ {{\text{ AUC}}_{{({\text{sc}})}} \times {\text{ DOSE}}_{{({\text{oral}})}} } \right] \, \times { 1}00\%$$

where AUC is the total area under the plasma INS concentration versus time curve.

All animals received a balanced diet and tap water ad libitum. Animals were carefully observed for the onset of any signs of toxicity and changes in body weight.

### The protective effect of BA-Al NPs on tissue damage

Before the text, diabetic rats were fasted overnight and remained free access to water. NS was administered orally to elucidate the normal shape of the tissue as negative control. Free form INS (50 IU/kg) and INS@ BA-Al NPs (50 IU/kg) were administered orally by intragastric administration, respectively. The SC injection of INS solution (5.0 IU/kg) were used as a positive control. The harvested organs (heart, liver, spleen, lung, and kidneys) were fixed in 10% paraformaldehyde after necropsy, and then washed, dehydrated, waxed, embedded, sectioned and sealed. The morphological characteristics of organs was performed by hematoxylin and eosin stain (H&E) and observed under microscope.

### Statistical analysis

All data are presented as mean ± standard deviation (SD). Pairs of groups was analyzed by the one-tailed Student’s test. A statistical difference was considered when significance *p* value was less than 0.05.

## Results and discussion

### Morphological and gelation characteristics of BA-Al NPs

We have been working on BA for a long time, and its self-assembly behavior has been previously reported [23]. In this work, we prepared BA-Al NPs by only one step without complex process. After mixing of BA and AlCl_3_ in an aqueous solution, the transparent orange-red hydrogel was obtained by heating in water bath (Fig. 1b and Additional file 1: Fig. S2).

The field-emission scanning electron microscopy (FESEM) and transmission electron microscopy (TEM) were employed to observe micromorphology of the nanoparticles. As shown in Fig. 1c and d, the obtained nanoparticles were uniform sphere with mean diameter of roughly 100 nm, and this result was consistent with that of dynamic light scattering (PDI = 0.28 ± 0.01) (Fig. 1e). The diameter of nanoparticles could be beneficial to transport through TJs [4]. Furthermore, zeta potential of BA-Al NPs dissolved in aqueous solution was − 47.93 ± 4.71 mV (Fig. 1f), indicating stability of nanoparticles which were less likely to aggregate and precipitate [28].

To further determine mechanical properties of the hydrogel, rheological analysis was experimented by testing the storage modulus (G′) and loss modulus (G″). Figure 1g indicated that G′ was consistently higher than G″ whether at 25 ℃ or 37 ℃, which revealed that temperature had little effect on the nanoparticles and possessed great performance in biomedical fields. In Fig. 1h, dynamic time sweep data showed that G′ was greater than G″ during the whole experiment of 600 s, and crossover point was not noticed when doing the sweep experiments. The rheology analysis displayed the stability and homogeneous of the fabricated hydrogel [29].

### Self-assembly mechanism of BA-Al NPs.

**Fig. 2:**
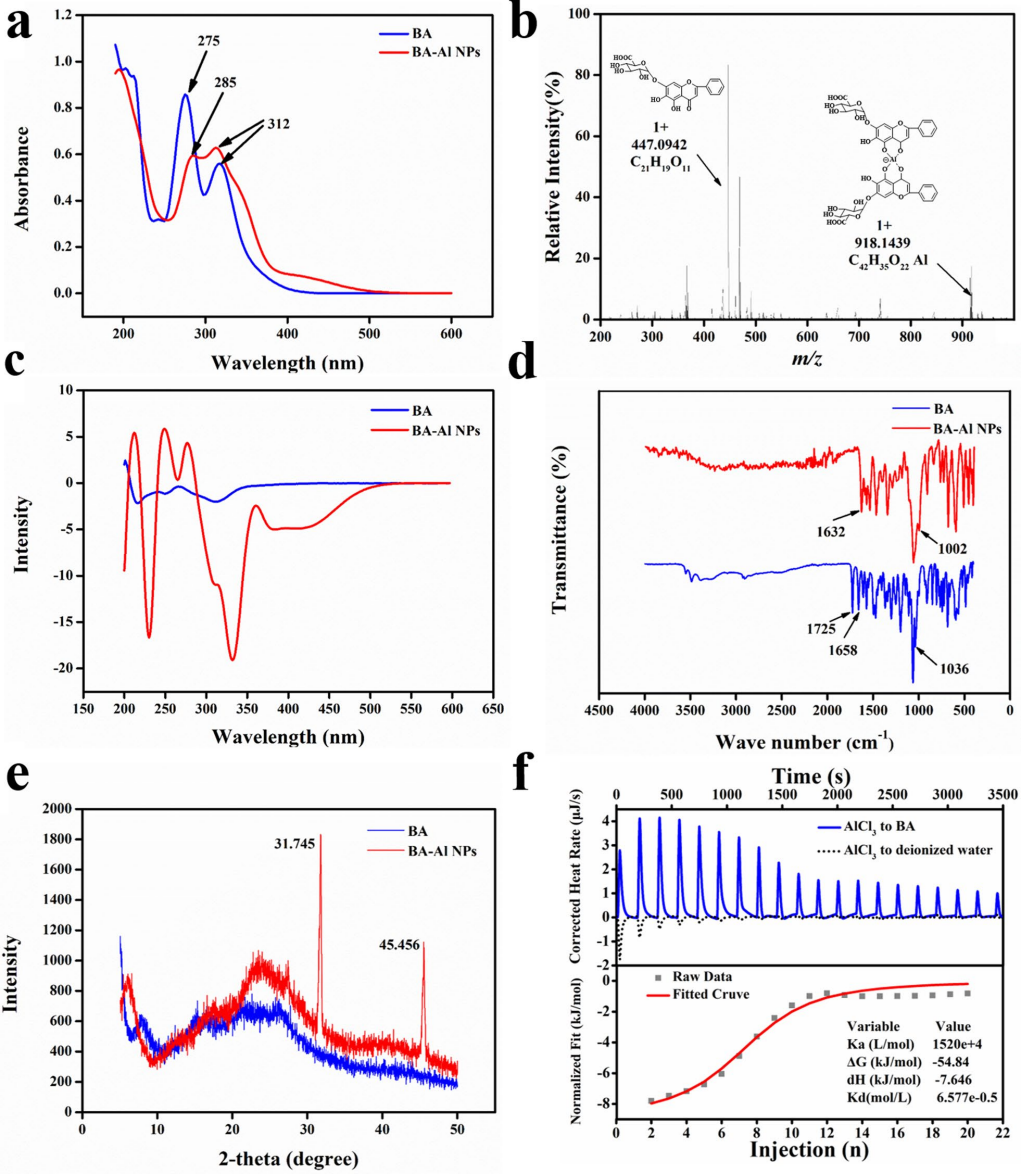
UV–vis spectrum of BA and BA-Al NPs (**a**); ESI-HRMS spectrum of BA and BA-Al NPs (**b**); CD spectra of BA and BA-Al NPs (**c**); FT-IR spectra of BA and BA-Al NPs (**d**); XRD pattern of BA and BA-Al NPs (**e**); ITC raw data and fitted curve of BA solution titrated by AlCl_3_ solution (**f**)

To thoroughly elucidate the structural properties of the BA-Al NPs, series of spectroscopy studies were implemented [30]. The Ultraviolet–visible (UV − vis) spectra of BA exhibited two significant absorption peaks at 275 nm (band II) and 312 nm (band I) (Fig. 2a). Compared with BA, there was an obvious bathochromic shift with band II on the characteristic absorption peak of BA-Al NPs (from 275 to 285 nm), but no change with band I was observed. The result indicated that 5-hydroxyl and 4-carbonyl group in A-ring of BA coordinated with Al^3+^ to form stable complex.

To obtain more intuitive complex fragment structure, high resolution mass spectrometry (HRMS) was performed. As shown in Fig. 2b, fragmentations of monomer (*m/z* 447.0942 [M + H] ^+^) and dimer (*m/z* 918.1439 [2M −2H + Al + H] ^+^) were found in ESI positive ion mode, which represented BA and BA-Al^3+^ complex respectively. The result showed that Al^3+^ attached to 4-carbonyl and 5-hydroxyl group of two BA and formed dimer through coordination bonds, which was consistent with the results of the UV–vis study. Meanwhile, the result of circular dichroism (CD) spectra (Fig. 2c) suggested that Al^3+^ transformed molecules from achiral to chiral component and induced the reinforcement of chiral features, which further indicated the formation of coordination bonds between Al^3+^ and BA [31].

Then Fourier transform infrared (FT-IR) spectra was determined to obtain more information on the nanoparticles. As shown in Fig. 2d, the spectra of unmodified BA displayed stretching vibration of the carbonyl group on glucuronic acid in 1725 cm^−1^, typical C_4_=O groups in 1658 cm^−1^ and C_5_-O groups in 1036 cm^−1^, respectively [32]. After forming nanoparticles, the main alterations in spectral absorption were as follows: down-field shift of C_4_ = O groups (from 1658 cm^−1^ to 1632 cm^−1^) and C_5_-O groups (from 1036 to 1002 cm^−1^) implied C_4_=O and C_5_-O binding sites might be occupied by Al^3+^. The peak of carbonyl group on glucuronic acid in the BA-Al NPs was disappeared, reflecting that there might be hydrogen bonding interaction.

Next, we applied X-ray diffraction (XRD) to further prove formation of coordination bonds and hydrogen bonds of the directly self-assembled nanoparticles in wide-angle patterns (Fig. 2e) [33]. Compared with BA, the diffractogram of BA-Al NPs showed two strong sharp reflections at 2θ = 31.745° (d = 2.8164 Å) and 2θ = 45.456° (d = 1.9937 Å). The sharp diffraction peaks were attributed to the partially ordered structure of the BA-Al NPs. The peak (d = 2.8164 Å) was due to the strong intermolecular hydrogen bond (COOH^−^) and the other peak (d = 1.9937 Å) was caused by the coordination bond (Al···O), respectively [34–36]. The XRD result indicated that incorporation of Al^3+^ rearranged the molecules to form coordination bonds and hydrogen bonds [37].

Isothermal titration calorimetry (ITC) was applied to detect the thermodynamic mechanism of the interaction between BA and AlCl_3_. As shown in Fig. 2f, AlCl_3_ solution (8 mM) was titrated into deionized water as benchmark to eliminate the interference of dilution heat, and AlCl_3_ solution (8 mM) titrated BA solution (1 mM) was set as an experimental group. The energy change data tested from ITC were summarized in Additional file 1: Tables S1 and S2. The titration of AlCl_3_ solution into deionized water showed a downward titration profile, indicating an endothermic dilution process. However, the titration of AlCl_3_ into BA revealed an utterly opposite trend and released much heat. The fitted S-shape curve generally presented strong interaction. Therefore, we confirmed that there was a strong interaction between BA and AlCl_3_. We also obtained binding thermodynamic parameters between them, and the negative value of *ΔG* (− 54.84 kJ·mol^−1^) indicated their binding reaction was spontaneous. *ΔH* (− 7.646 kJ·mol^−1^) and *TΔS* (− 16.617 kJ·mol^−1^) indicated their binding was enthalpy-driven reaction, which meant the existence of hydrogen bonds [38]. All of the binding thermodynamic parameters illustrated a chemical reaction rather than physical combination in the well-organized self-assembly process [23].

**Scheme 1:**
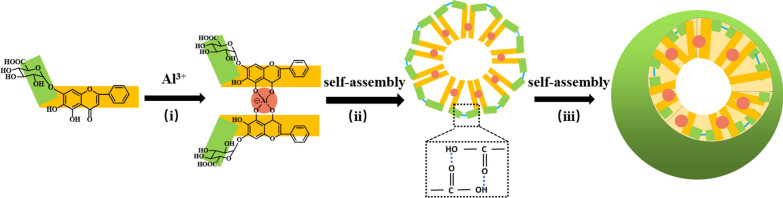
A hypothetical mechanism of self-assembly formation of the spherical BA-Al NPs. (i) The carbonyl and hydroxy groups on the two BA self-assembled to form dimer by binding Al^3+^. (ii) The dimers are connected by intermolecular hydrogen bonds. (iii) The dimers cross-linked to form three-dimensional spherical nanoparticles

In summary, all of UV–vis, HRMS, CD and FT-IR could infer that two BA connected with Al^3+^ to form dimer through coordination bonds. Both FT-IR and XRD further suggested not only coordination bonds, but hydrogen bonds were formed in the process of self-assembly. Furthermore, ITC demonstrated spontaneous reactions in the binding process were a chemical rather than physical reaction. Based on the above experimental results, as shown in Scheme 1, two BA molecules self-assembled to form a one-dimensional dimer through coordinate bond with Al^3+^. Dimers then were connected to form three-dimensional spherical nanoparticles through intermolecular hydrogen bonds.

### In vitro release of INS from INS@BA-Al NPs

**Fig. 3:**
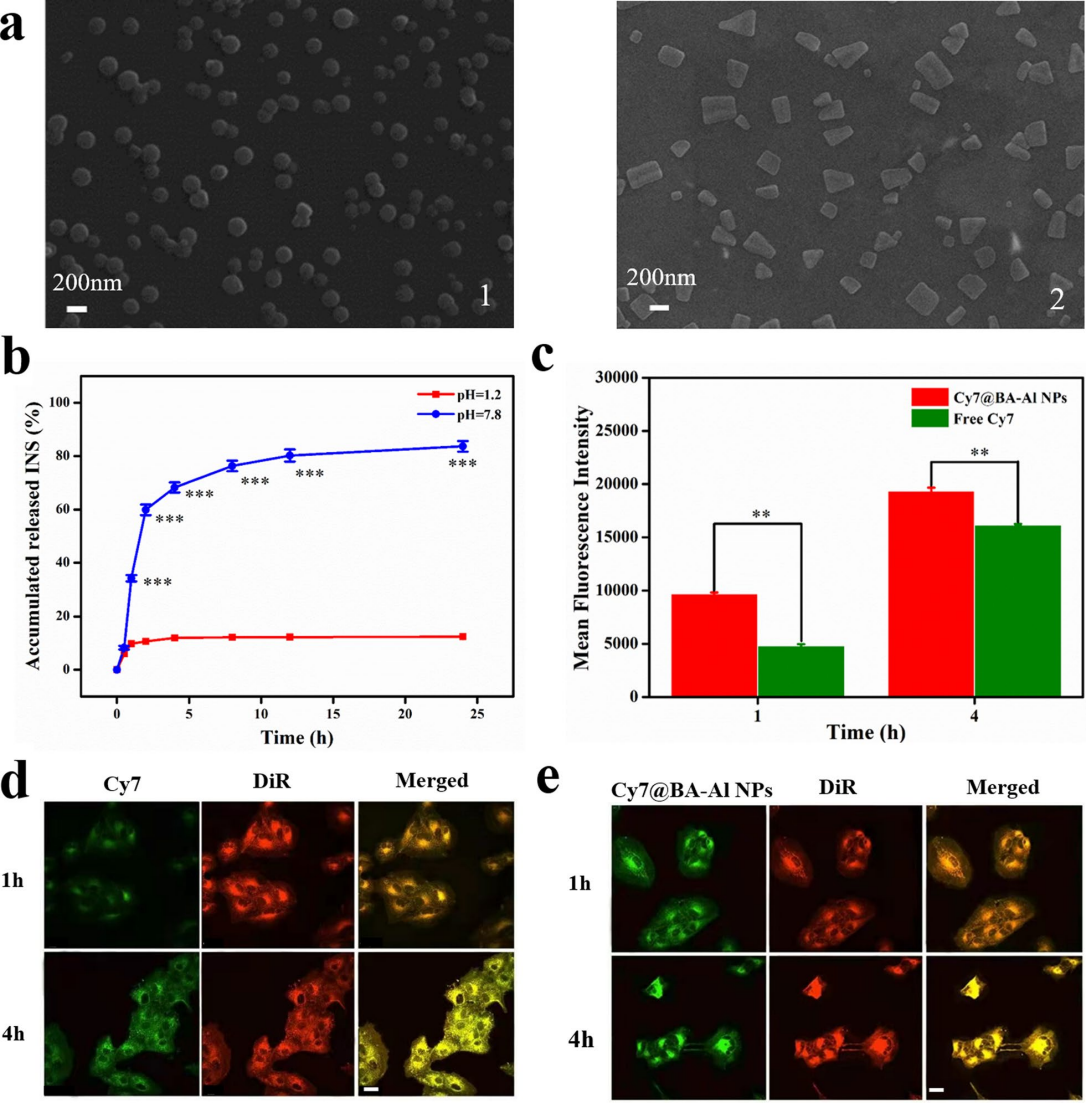
SEM images of INS@BA-Al NPs at (1) acidic condition (pH = 1.2) and (2) alkaline condition (pH = 7.8) (**a**); In vitro INS release of INS@BA-Al NPs at different pH conditions (n = 3, error bars = SD) *** = *p* < 0.001 (**b**); Flow cytometric analysis and quantitative results of the cells treated with free-form Cy7 and Cy7@BA-Al NPs at 1 h and 4 h, (n = 3, error bars = SD), ** = *p* < 0.01 (**c**); Confocal laser scanning microscopy images of MDCK cells after incubation with free-form Cy7, DiR for 1 h and 4 h. The scale bar = 50 μm. Green and red signals correspond to Cy7 and DiR, respectively (**d**); Confocal laser scanning microscopy images of MDCK cells after incubation with Cy7@BA-Al NPs, DiR for 1 h and 4 h. The scale bar = 50 μm (**e**)

To further explore the drug transport capacity of the prepared carrier, in vitro release behavior of BA-Al NPs was studied under artificial gastric juice (pH = 1.2) and artificial intestinal juice (pH = 7.8) at 37 °C, respectively. As displayed in Fig. 3A, the morphological of the INS@BA-Al NPs was integrated under acidic condition (Fig. 3a1), while under alkaline condition, INS@BA-Al NPs began to dissolve (Fig. 3a2). The main reason may be that two BA molecules were tightly bound by hydrogen bonds in an acidic environments. In contrast, INS@BA-Al NPs became loose as destruction of intermolecular hydrogen bonds in an alkaline environment. Therefore, the BA-Al NPs has potential in drug delivery system due to its integrity can be controlled by changing pH circumstances.

We then evaluated controlled releasing function of INS@BA-Al NPs under different pH values. In Fig. 3b, the cumulative solubilization curves showed that the drug release rate was 12.4% of encapsulation INS by BA-Al NPs under the artificial gastric juice (pH = 1.2), whereas 83.7% INS was released from BA-Al NPs under the intestinal gastric juice (pH = 7.8). This demonstrated that the BA-Al NPs was pH-sensitive and could control the release of INS under different pH conditions. Considering dramatic pH fluctuations in the gastrointestinal tract, this pH-responsive property of BA-Al NPs is beneficial for retaining the stability of INS in stomach acid and controlling release of INS in the intestines.

### Cellular uptake analysis

Confocal laser scanning microscopy (CLSM) was performed to detect MDCK cells incubated with BA-Al NPs to gain further insight into cellular adhesion and uptake of the nanoparticles [39]. As Fig. 3d and 3e showed, the intense green fluorescence Sulfo-Cyanine7 (Cy7) confirmed that BA-Al NPs was sufficiently stuck to MDCK cells and cytomembrane was labelled by red DiR iodide (DiR), and the yellow signal was overlap of Cy7 and DiR fluorescence. There was more significant yellow signal intensity in the cytomembrane of MDCK cells after 1 h incubated with Cy7@BA-Al NPs than free-form Cy7. As the incubation time extending to 4 h, more Cy7@BA-Al NPs adhered to cells, resulting in an increase in fluorescence intensity. We further used flow cytometry to research cellular uptake efficient of MDCK cells treated with Cy7@BA-Al NPs and free-form Cy7, Fig. 3c indicating that Cy7@BA-Al NPs possessed higher internalization ability in 1 h and 4 h. Collectively, this phenomenon manifested that BA-Al NPs as oral delivery vehicle could be adhered around and taken into cells.

### Biodistribution and mucoadhesion of BA-Al NPs

**Fig. 4:**
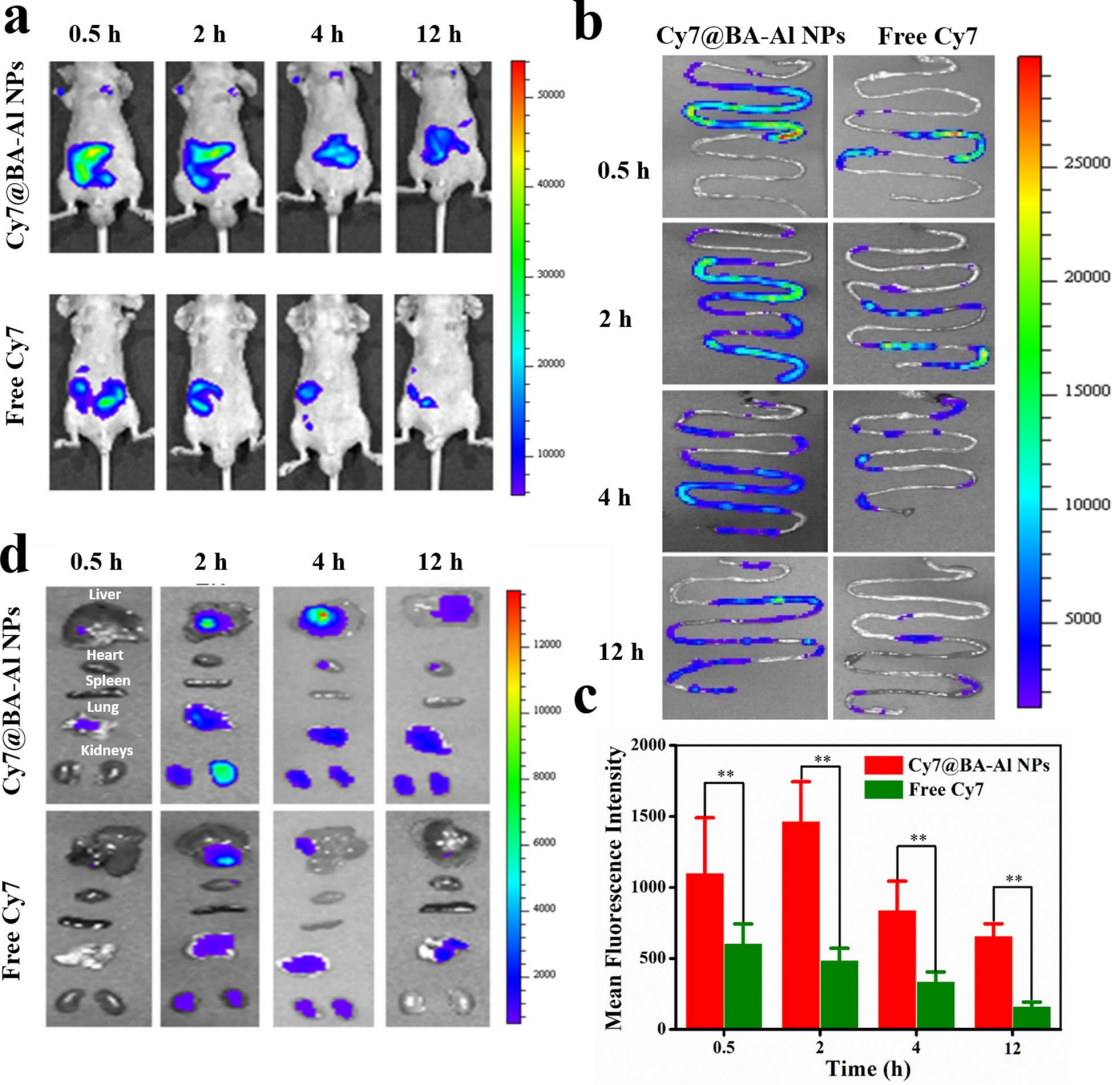
Whole-body fluorescence images of mice at different time points after being treated with free-form Cy7 and Cy7@BA-Al NPs via oral administration (**a**); Fluorescence images of in vitro intestine at different time points after mice being treated with free-form Cy7 and Cy7@BA-Al NPs via oral administration (**b**); Flow cytometric analysis and quantitative results of in vitro intestine at different time points after mice being treated with free-form Cy7 and Cy7@BA-Al NPs via oral administration, (n = 3, error bars = SD). ** = *p* < 0.01 (**c**); Fluorescence images of organs at different time points after mice being treated with free-form Cy7 and Cy7@BA-Al NPs via oral administration (**d**)

Besides drastic changes in pH, the rapid removal at intestine is an important biological obstacle [40]. To investigate the effect of mucoadhesion of free-from Cy7 and Cy7@BA-Al NPs, they were orally administered to mice, and the fluorescent signal was observed by a small animal imaging system in vivo. From the whole body's fluorescence signal (Fig. 4a), mice administered with Cy7@BA-Al NPs displayed more sustained fluorescence signal than free-form Cy7. As shown in Fig. 4b, with the time extended to 12 h, the Cy7@BA-Al NPs group still had continuous fluorescence in intestine, while Cy7 group had almost no fluorescence. The time-dependent change of fluorescence intensity was shown in Fig. 4c, the fluorescence signal stayed longer time at intestine of mice treated with Cy7@BA-Al NPs compared to free-form Cy7. The result suggested that mucoadhesive hydrogel could prolong the retention time of agent in intestine. In addition, as showed in Fig. 4d, organs were retrieved for *ex-vivo* imaging. The Cy7@BA-Al NPs fluorescence intensity at liver and kidney were higher than free Cy7 evidently, indicating systemic absorption and metabolism of Cy7@BA-Al NPs [41].

### Mechanism of BA-Al NPs actions

**Fig. 5:**
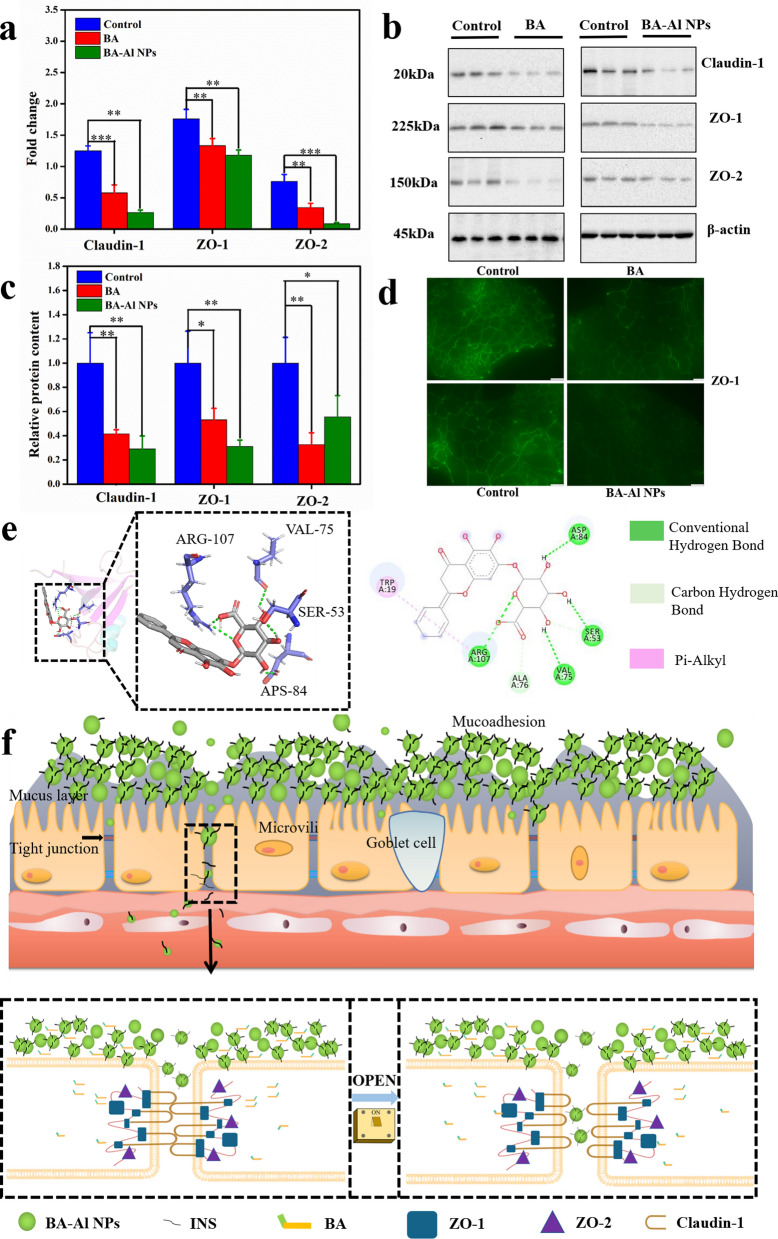
Gene expression levels of Claudin-1, ZO-1 and ZO-2 mRNAs in BA and BA-Al NPs-treated Caco-2 cells. Caco-2 cells treated with DMEM medium were used as a blank control * = *p* < 0.05, ** = *p* < 0.01, *** = *p* < 0.001 (**a**); Representative western blot of Claudin-1, ZO-1 and ZO-2 proteins in BA and BA-Al NPs- treated Caco-2 cells (**b**); Quantification of Claudin-1, ZO-1 and ZO-2 proteins using Image J. * = *p* < 0.05, ** = *p* < 0.01, *** = *p* < 0.001 (**c**); Immunofluorescence staining of TJ protein ZO-1 in BA and BA-Al NPs-treated Caco-2 cells (**d**); Molecular docking analysis of interaction between BA and 2h3m (crystal structure of ZO-1 PDZ1) (**e**); Schematic illustration showing the mechanism and consequence of BA-Al NPs mediated TJs modulating between intestinal epithelial cells (**f**)

Tight junctions (TJs) are the major obstacle to INS absorption in paracellular pathway. The integrity of TJs is crucial in expounding the mechanism of BA-Al NPs-induced TJs opening [42]. The protein–protein interaction between claudins (claudin-1) and ZO scaffolding proteins (ZO-1, ZO-2) is the major basis of integrity of TJs [43–45]. In this study, the mRNA and protein expression level of TJs proteins was tested by real-time (RT)-PCR and western blot (WB) analysis, respectively. The expression of TJs protein of Caco-2 cells after the administration of BA and BA-Al NPs was researched at both gene and protein level. The result demonstrated BA and BA-Al NPs could down-regulate or redistribute TJs proteins (Fig. 5a–c). In addition, as shown in Fig. 5d, the result of immunofluorescence staining also showed that expression of ZO-1 proteins of BA and BA-Al NPs treated Caco-2 cells was significantly weakened and discontinuous, especially with respect to controls.

We hypothesized that BA-Al NPs could cause the TJs proteins to be downregulated, which might be attributed to the important role played by BA. Under the assumption, we further used molecular docking to study the potential interaction between BA and protein domain. The result showed that BA bound to 2h3m of ZO-1 simply and stably (*△G* bind = − 7.7), which might be what causes the nanoparticles to downregulate the TJs proteins. The hydrogen bond and pi-alkyl are mainly interaction between BA and the residues of 2h3m (Fig. 5e). In addition, the expression of claudin-1, ZO-1 and ZO-2 decreased after the administration of BA compared with the control group at both gene and protein level (Fig. 5a–c). The result was consistent with previous report demonstrating that BA down-regulated or redistributed TJs proteins to influence the integrity of TJs [17, 27].

These findings suggested that expression of TJs-related genes and proteins of Caco-2 cells induced by BA-Al NPs were related to the down-regulation and redistribution of TJs protein by BA. Interestingly, under the action of self-assembly, the bioavailability of BA in BA-Al NPs was increased, which may cause the down-regulation of most genes and proteins was more obvious by BA-Al NPs. BA-Al NPs could modulate epithelial barrier in human intestinal Caco-2 cells and has a potential to become an idea drug absorption enhancer through paracellular pathway (Fig. 5f).

### In vivo pharmacokinetics and pharmacodynamics of INS@BA-Al NPs.

**Fig. 6:**
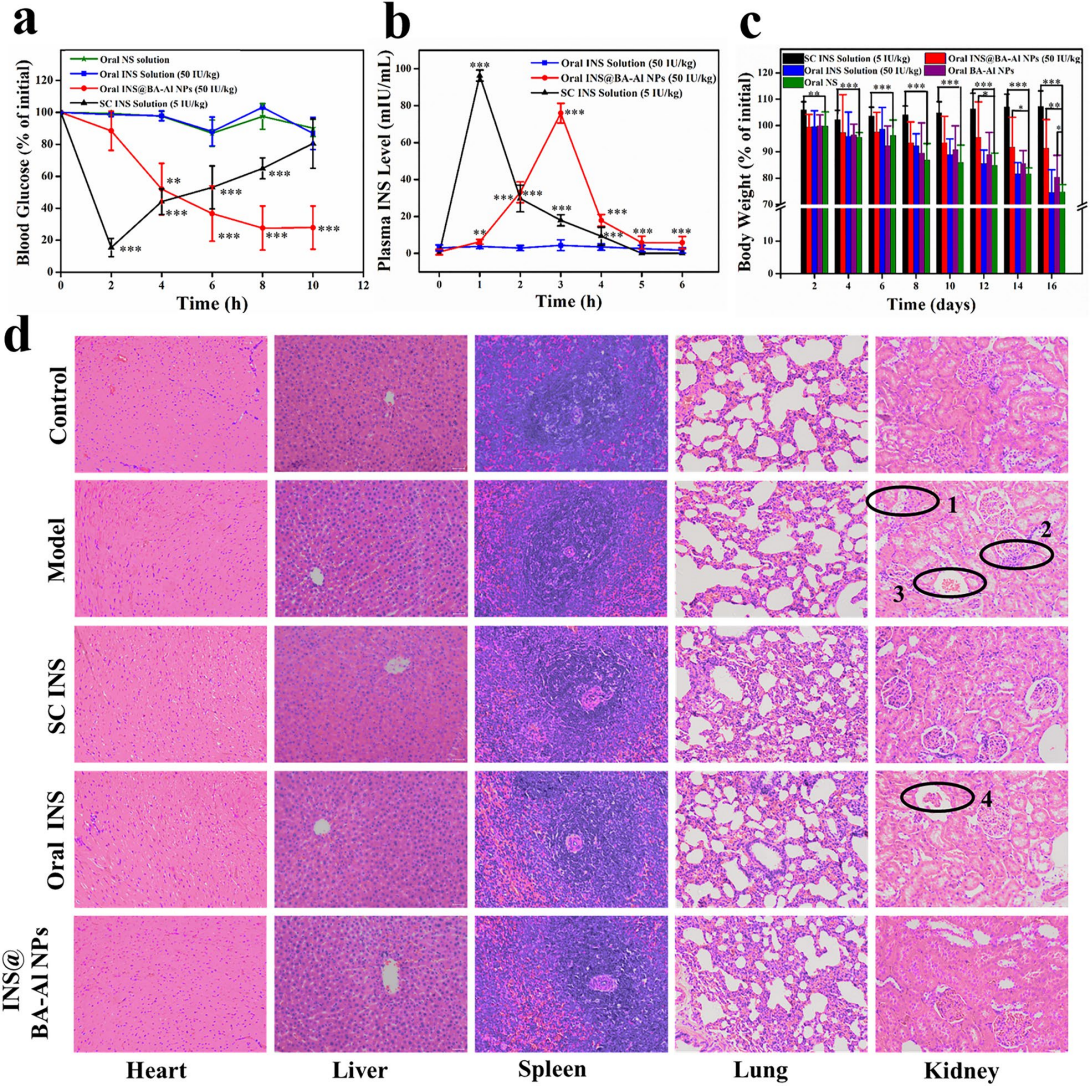
Hypoglycemic effect after oral administration in rats of normal saline (NS), free-form INS, INS@BA-Al NPs and SC administration of INS solution (n = 5, error bars = SD) (**a**); Plasma INS level versus time profiles of diabetic rats treated with oral administration of free-form INS solution, INS@BA-Al NPs and SC administration of INS solution (n = 5, error bars = SD) (**b**); Changes in body weight for 16 days (n = 3, error bars = SD). * = *p* < 0.05, ** = *p* < 0.01, *** = *p* < 0.001 (**c**); The photomicrographs of major organs (H&E staining, magnification 200 ×) of normal group, model group, diabetic rats treated with INS@BA-Al NPs group and free-form INS group, SC INS group for 16 days. The scale bar = 50 μm (**d**)

To test our hypothesis and decipher regulation of blood glucose level of INS@BA-Al NPs after oral administration, in vivo hypoglycemic effect was investigated on type I diabetic rats. The blood glucose level-time profiles following administration of various INS formulations to diabetic rats were shown in Fig. 6a. As expected, no hypoglycemic effect was observed after diabetic rats orally administrated free-form INS, implying a poor hypoglycemic effect of insulin without an appropriate carrier. Subcutaneous (SC) injection of the insulin markedly reduced the blood glucose level in 0–2 h, and subsequently its blood glucose level returned to initial level. INS@BA-Al NPs showed a remarkable reduction in blood glucose level and prolonged hypoglycemic time. This result suggested oral administration of INS@BA-Al NPs could steadily control the blood glucose and displayed an apparent reduction in blood glucose level.

The corresponding plasma INS concentration at different time were researched to evaluate relative bioavailability of INS [46]. As shown in Fig. 6b, in the group orally administered with the free-form INS, the amount of plasma INS was hard to detect. The group subcutaneously treated with free-form INS showed a maximum serum INS concentration at 1 h post-administration. The INS@BA-Al NPs revealed a maximum concentration of plasma INS at 3 h post-administration which meant prolonged circulation time. The area under the concentration–time curve (AUC _(0–6 h)_) of group orally treated with INS@BA-Al NPs was 75.93 ± 5.34 *μ*IU·h·mL^−1^, which corresponded to the relative bioavailability of INS found to be 8.88 ± 1.05%. These results demonstrated that the BA-Al NPs enhanced bioavailability of INS.

Simultaneously, we monitored body weight of rats to exam the therapeutic efficacy and potentially adverse effect of BA-Al NPs. As shown in Fig. 6c and Additional file 1: Table S3, distinct body weight changes were revealed in four different groups. The diabetic rats in normal saline (NS) and free-form INS group displayed a sharp decrease in body weight, which revealed serious side effects induced by diabetic. While the INS@BA-Al NPs and BA-Al NPs treated groups unveiled a minor decline of body weight loss, showing therapeutical effect and reduced system toxicity.

The histologic sections stained with hematoxylin and eosin (H&E) were carried out to estimate pharmacodynamic and long-term toxicity of INS@BA-Al NPs. As shown in Fig. 6d1, d2 and d3, the kidney in the model group showed edema of renal tubular epithelial cells, infiltration of inflammatory cells in renal interstitium, and renal tubular cell cast. The oral free-form INS group showed glomerular atrophy, indicating that oral INS had no protective effect on the kidney of diabetic rats (Fig. 6d4). In addition, there is little tissue injury or infiltration could be observed in the organs of the SC INS and INS@BA-Al NPs group, indicating that SC INS and INS@BA-Al NPs has a protective effect on the kidney. In addition, compared with the control group, no significant pathological feature was founded in the liver and heart of the diabetic rats treated with INS@BA-Al NPs. These results also highlighted the superiority of BA-Al NPs as nanocarriers to delivery INS.

### Biosafety evaluation of BA-Al NPs

**Fig. 7:**
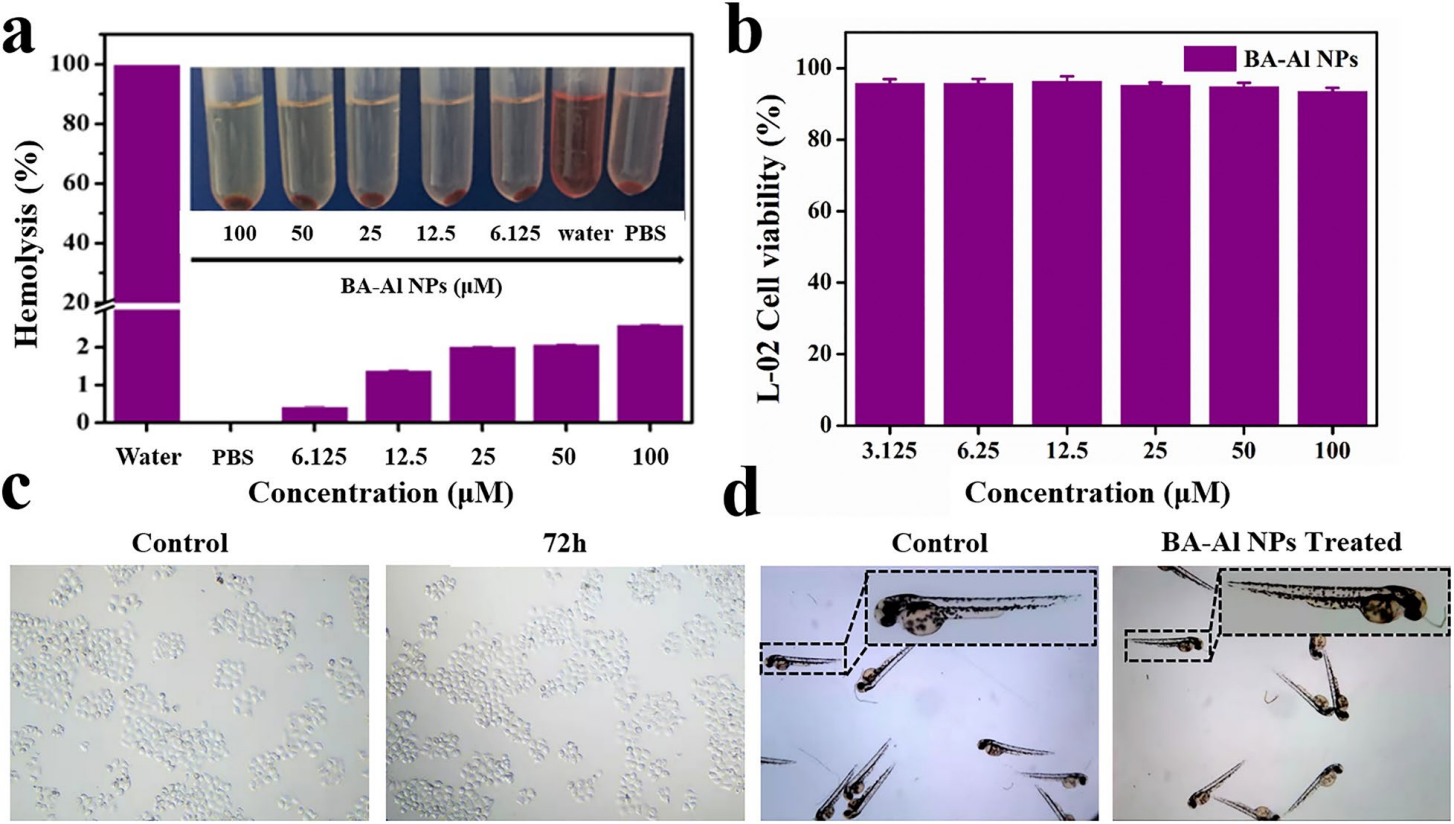
Hemolysis assay of red blood cells treated with deionized water, PBS, and different concentrations of BA-Al NPs (**a**); Cell viability of L-02 cells incubated with BA-Al NPs for 72 h (**b**); Image of L-02 cells (**c**); Image of zebrafish of larvae treated with Holtfreter’s solution and BA-Al NPs for 72 h (**d**)

As a newly discovered drug delivery carrier for oral administration, the biosafety of BA-Al NPs was of vital importance. The effective therapeutic dose of aluminum ions was still far less than the heavy metal guidelines of the United States Pharmacopoeia (50,000 μg/day) and experiments were used to prove its safety. Hemolysis assay, cell cytotoxicity test and zebrafish model were performed in vitro and in vivo respectively. Hemolytic assays result suggested that even the concentration of BA-Al NPs as high as 100 μM, it had no obvious hemolytic activity in the rats’ red blood cells (Fig. 7a). The hemolysis rate of BA-Al NPs was far lower than the internationally recognized standard (5%), indicating its good biocompatibility. The result showed that BA-Al NPs had potential application in blood contact [47].

To further evaluate biosafety of BA-Al NPs, L-02, MIHA and HEK-293 T cells were carried out to test the cytotoxicity by an MTT assay [48]. As shown in Fig. 7b, c and Additional file 1: Fig. S3, cell viability of BA-Al NPs was over 90% even after incubated with BA-Al NPs at the highest concentration of 100 μM for 72 h, which revealed the BA-Al NPs had no adverse effect on L-02, MIHA and HEK-293 T cells.

Zebrafish can be used as an in vivo model to test biosafety due to its feasibility and high fecundity. We employed zebrafish to evaluate the biosafety of BA-Al NPs at the concentrations rang of 6.125–100 μM. In the experiment, healthy 1- day old zebrafish larvae were cultured with BA-Al NPs for 72 h. As shown in Fig. 7d, all zebrafish survived in both groups during the whole test, demonstrating BA-Al NPs had little toxicity on the growth of zebrafish larvae. All the results suggested that the BA-Al NPs had good biosafety and biocompatibility.

## Conclusion

In this study, we explored distinctive self-assembled BA-Al NPs, which induced by coordination bonds and hydrogen bonds. To increase the bioavailability of INS, the advantages offered by BA-Al NPs are as follows: (i) The pH-sensitivity of the nanoparticles ensured the stability of the drug under gastric acid conditions and released in the intestine; (ii) BA-Al NPs with gelatinous properties allowed for gradual and continuous release of insulin, which prolonged the residence time in intestine; (iii) BA-Al NPs disassembled and released BA to downregulate the TJs proteins expression and further improve the absorption of INS to maintain glucose homeostasis through modulating TJs. In vivo pharmacological activity showed INS@BA-Al NPs could regulate blood glucose level and prolong circulation time. As a prospect, strategy of self-assembly nanoparticles fabricated by SMNPs could be a new field for INS delivery, and a variety of active SMNPs self-assembled with metal ions can be used to various drug delivery system for the good biocompatibility and effective treatment.

## Supplementary Information

**Additional file 1: Experiment methods**. Materials; INS structural stability under different pH conditions; Molecular docking studies; Cell culture; In vitro toxicity assessment by cell viability; Hemolysis assay; Cytotoxicity evaluation by zebrafish assay; **Table S1**. Energy changes data for the interactions obtained from ITC. **Table S2**. Binding thermodynamics of AlCl3 into BA. **Table S3**. Changes in body weight of rats treated with INS@BA-Al NPs, oral INS solution, BA-Al NPs, NS and normal rats. **Figure S1**. (a-c) Far−UV CD spectra of INS for period of 36h. (d-f) Fluorescence spectra of INS for period of 36h. **Figure S2**. Morphology of BA-Al NPs. **Figure S3**. (a) Cell viability of MIHA cells incubated with BA-Al NPs for 72 h. (b) MIHA cells image of the control group and the 72 h group treated with BA-Al NPs for 72 h. (c) Cell viability of HEK-293T cells incubated with BA-Al NPs for 72 h. (d) HEK-293T cells image of the control group and the 72 h group treated with BA-Al NPs for 72 h.

## Data Availability

All data generated or analysed during this study are included in this article and its additional file.
