# Supplementary material for: Multi-functional self-assembly nanoparticles originating from small molecule natural product for oral insulin delivery through modulating tight junctions
Source: J Nanobiotechnology. 2022 Mar 5;20:116. doi: 10.1186/s12951-022-01260-9 (PMC8898475; doi:10.1186/s12951-022-01260-9)
Supplement: Supplementary file 1 — Additional file 1: Experiment methods. Materials; INS structural stability under different pH conditions; Molecular docking studies; Cell culture; In vitro toxicity assessment by cell viability; Hemolysis assay; Cytotoxicity evaluation by zebrafish assay; Table S1. Energy changes data for the interactions obtained from ITC. Table S2. Binding thermodynamics of AlCl3 into BA. Table S3. Changes in body weight of rats treated with INS@BA-Al NPs, oral INS solution, BA-Al NPs, NS and normal rats. Figure S1. (a-c) Far−UV CD spectra of INS for period of 36h. (d-f) Fluorescence spectra of INS for period of 36h. Figure S2. Morphology of BA-Al NPs. Figure S3. (a) Cell viability of MIHA cells incubated with BA-Al NPs for 72 h. (b) MIHA cells image of the control group and the 72 h group treated with BA-Al NPs for 72 h. (c) Cell viability of HEK-293T cells incubated with BA-Al NPs for 72 h. (d) HEK-293T cells image of the control group and the 72 h group treated with BA-Al NPs for 72 h. [file 12951_2022_1260_MOESM1_ESM.docx]

**Additional file**

**Multi-functional Self-Assembly Nanoparticles Originating from Small Molecule Natural Product for Oral Insulin Delivery through Modulating Tight Junctions**

Xiaohui Jia‡, Zhihua Yuan‡, Yuqin Yang, Xuemei Huang, Nana Han, Xiaojing, Liu, Xiaoyu Lin, Tao Ma, Bing Xu, Penglong Wang*, Haimin Lei*

School of Chinese Pharmacy, Beijing University of Chinese Medicine, Beijing 102488, P. R. China

‡ These authors contributed equally.

*Corresponding authors. Tel./fax: +86 10 53912129.

E-mail addresses: wpl581@126.com (Penglong Wang), hm_lei@126.com (Haimin Lei).

**Materials**

Baicalin (C_21_H_18_O_11_, MW 446.36) was purchased from Chengdu Pufei De Biotch Co., Ltd (Chengdu, China). AlCl_3_ was purchased from Chengdu Pufei De Biotch Co., Ltd (Chengdu, China). NaOH was obtained from Chengdu Pufei De Biotch Co., Ltd (Chengdu, China). INS (27.4 IU/mg, Sigma-Aldrich, St. Louis, MO, USA). Fetal bovine serum (FBS) and Dulbecco’ modified Eagle’ medium (DMEM) were obtained from Gibco Life Technologies (AG, Switzerland). 3-(4, 5-Dimethyl-thiazol-2-yl)-2,5-diphenyl tetrazolium bromide (MTT) was purchased from Beyotime Institute of Biotechnology and used without further purification. Sulfo-Cyanine7 (Cy7) and DiR iodide (DiR) was purchased from Sigma-Aldrich (Missouri, America). All of the glassware were dried at 120 °C overnight and cooled under vacuum before use. BCA protein concentration assay kit was purchased from Biorigin (Beijing China). Streptozotocin (STZ) was purchased from rhawn (Shanghai China).

**INS structural stability under different pH conditions**

The secondary structure of insulin was analyzed by far-UV CD spectroscopy. Spectra were recorded on Chirascan V100 (Applied Photophysics, UK). The temperature was kept constant at 25 ± 1 ◦C, and spectra were measured from 200 - 300 nm. The samples analyses were obtained by dissolution of the NPs in deionized Water, and INS concentrations were normalized to 0.43 mg/mL. In addition, fluorescence spectra was also used to further support the results of CD. The emission spectrum of INS was determined at 276 nm, which was the maximum excitation wavelength of INS.

**Molecular docking studies.**

Molecular docking studies were used to assess the probability and stability of BA binding with ZO-1. After downloading the PBD file of the protein 2h3m, which is crystal structure of ZO-1 PDZ1, from the PDB database (http://www.rcsb.org). The file was imported into AutoDock 4.2.6. The file was select the Assign AD4 Type to be stored in PDBQT format after water removal and hydrogenation. The mol file of BA was obtained from chemical book database (https://www.chemicalbook.com). Chem3D 17.1 was used to transform the format from mol to mol_2_ and minimized the energy. The resulting file was imported into AutoDock 4.2.6 software to hydrogenate. The central node of the ligand molecule was set to rotatable and output in PDBQT format. AutoDock was used to create grid Spacing parameter file (X Center = 11.778, Y Center = 22.778, Z Center = 15.333, X-dimension = 38, Y-dimension = 60, Z-dimension = 56, box Spacing = 0.375), after the generation of docking parameter file (DPF) and result in AutoDock4.2.6, Discovery Studio 2016 Client was performed for optimization, and Pymol was used to draw.

**Cell culture.**

Caco-2, HEK293T and MIHA cell lines were purchased from American Type Culture Collection (Manassas, VA, USA). L-02 and MDCK were obtained from the Chinese Academy of Medical Sciences & Peking Union Medical College. Caco-2, HEK293T and MDCK cell lines were grown at 37 °C with 5% CO_2_ in a humidified incubator in Dulbecco’s modified Eagle’s medium (DMEM) high glucose (Gibco, Thermo Fisher Scientific) supplemented with 10% (v/v) fetal bovine serum (Gibco, Thermo Fisher Scientific) and 1% (v/v) penicillin–streptomycin (Gibco, Thermo Fisher Scientific). MIHA and L-02 cell lines were grown at 37 °C with 5% CO_2_ in a humidified incubator in (RPMI) 1640 Medium high glucose (Gibco, Thermo Fisher Scientific) supplemented with 10% (v/v) fetal bovine serum (Gibco, Thermo Fisher Scientific) and 1% (v/v) penicillin–streptomycin (Gibco, Thermo Fisher Scientific). Unless specified, the medium was refreshed every two days.

***In vitro* toxicity assessment by cell viability.**

The MTT assay was performed on HEK293T, MIHA and L-02 cells to measure cytotoxicity of BA-Al NPs. Cells were cultured in 96-well plates, and the density in each well was 4×10^3^/mL. After seeded for 24h, 100 μL medium containing different concentrations of nanoparticles (6.125 μM, 12.5 μM, 25 μM, 50 μM, 100 μM) were added to wells. After 72 h of incubation, MTT (20 μL, 5 mg/mL) was added to each well. Then the plate was incubated for 4h in an incubator (BPH-9082, Scientific Instrument Co., LTD, China) with a constant temperature of 37 ℃ and saturated humidity of 5% CO_2_ for 4 h. Discard the supernatant and add the 150 μL formazan dissolved in DMSO into each well and shock it away from light for 5 min. Optical density (OD) value was read in 490 nm wavelength using microplate reader (BIORAD 550 spectrophotometer, Biorad Life Science Development Ltd., Beijing, China). Wells without drugs were used to be blanks. The cell viability (%) was calculated in the following Equation (2):

cell viability = (Sample group OD-Blank group OD) / (Control group OD-Blank group OD) × 100% (2)

**Hemolysis assay.**

Red blood cells were taken from fresh rat blood. SPECTROstar Nano (BMG LABTECH, Offenburg, Germany) were used to measure the absorption of supernatant was read at 570 nm by microplate reader and take the average of three calculations. The positive control was water and the negative control was PBS. The Equation (3) was used to calculate the hemolysis rate:

Z = (D_t_ - D_nc_) / (D_pc_ - D_nc_) × 100% (3)

where Z is hemolytic rate, D_t_ is hemolysis absorbance of the experimental group, D_nc_ is hemolysis absorbance of negative control group, D_pc_ is hemolysis absorbance of positive control group.

**Cytotoxicity evaluation by zebrafish assay.**

Zebrafish model was performed to evaluate toxicity *in vivo*. The assays were carried out in 12-well plates. Embryo culture medium was prepared according to the zebra fish manual. BA-Al NPs was dissolved in the embryo culture medium to produce 1 mg/mL solution, and then dissolved in the culture water to prepare 6 concentration gradients. The wells of plates were filled with 20 randomly selected day-old juvenile fish treat with sample of BA-Al NPs as experimental group and the culture water without nanoparticles as control group. After continuous administration for 72 h, sufficient oxygen was provided, and new culture water was replaced every day. The 12-well plate was wrapped with tin foil to prevent the formation of melanin in zebrafish. The 12-well plate was cultured in a 27 ℃ incubator. The zebrafish were observed under a stereoscopic microscope 72 h later, and the morphological changes of zebrafish were recorded.

**Table S1** Energy changes data for the interactions obtained from ITC.

| Number | AlCl_3_ to BA  [*μ*J] | AlCl_3_ to water  [*μ*J] | Correction  [*μ*J] |
| --- | --- | --- | --- |
| 1 | -87.33 | 54.66 | -141.99 |
| 2 | -129.80 | 26.21 | -156.01 |
| 3 | -131.50 | 18.01 | -149.51 |
| 4 | -130.00 | 13.43 | -143.43 |
| 5 | -124.50 | 10.12 | -134.62 |
| 6 | -113.20 | 7.77 | -120.97 |
| 7 | -91.97 | 5.66 | -97.63 |
| 8 | -67.64 | 4.80 | -72.44 |
| 9 | -44.49 | 3.77 | -48.26 |
| 10 | -29.05 | 2.74 | -31.79 |
| 11 | -17.48 | 2.10 | -19.58 |
| 12 | -15.26 | 0.77 | -16.03 |
| 13 | -17.77 | 0.71 | -18.48 |
| 14 | -19.21 | 0.73 | -19.94 |
| 15 | -19.13 | 0.70 | -19.83 |
| 16 | -19.79 | -0.06 | -19.73 |
| 17 | -18.96 | -0.54 | -18.42 |
| 18 | -18.81 | -0.01 | -18.80 |
| 19 | -18.09 | -0.73 | -17.36 |
| 20 | -17.38 | -0.99 | -16.39 |

**Table S2** Binding thermodynamics of AlCl_3_ into BA.

| No. | Δ*H*  [kJ mol^-1^] | *-T*Δ*S*  [kJ mol^-1^] | Δ*G*  [kJ mol^-1^] | *Kd*  [mol/L] | *Ka*  [L/mol] |
| --- | --- | --- | --- | --- | --- |
| AlCl_3_ to BA | -7.646 | -16.617 | -54.84 | 6.577×10^-0.5^ | 1.520×10^4^ |

**Table S3** Changes in body weight of rats treated with INS@BA-Al NPs, oral INS solution, BA-Al NPs, NS and normal rats.

| Times (days) | SC INS Solution  [5IU kg^-1^] | INS@BA-Al NPs  [50IU kg^-1^] | Oral INS Solution  [50IU kg^-1^] | BA-Al NPs | NS |
| --- | --- | --- | --- | --- | --- |
| 2 | 105.99±3.02 | 99.44 ± 4.74 | 99.59 ± 6.01 | 99.88 ± 4.14 | 99.74 ± 5.41 |
| 4 | 102.15±3.60 | 97.36 ± 14.37 | 95.96 ± 9.11 | 96.35 ± 4.12 | 95.43 ± 1.84 |
| 6 | 103.60±3.36 | 97.45 ± 7.52 | 98.57 ± 8.29 | 92.30 ± 7.53 | 96.18 ± 5.89 |
| 8 | 104.05±3.36 | 93.50 ± 7.86 | 92.25 ± 4.58 | 89.55 ± 11.43 | 86.87 ± 6.22 |
| 10 | 104.86±4.13 | 93.51 ± 9.94 | 88.88 ± 5.99 | 90.82 ± 9.06 | 85.96 ± 6.59 |
| 12 | 106.32±5.89 | 95.47 ± 13.47 | 85.56 ± 5.09 | 88.91 ± 8.45 | 84.84 ± 4.67 |
| 14 | 107.09±4.78 | 91.76 ± 11.34 | 81.68 ± 4.20 | 85.48 ± 5.00 | 81.58 ± 2.27 |
| 16 | 107.29±5.83 | 91.34 ± 10.90 | 74.56 ± 8.61 | 80.43 ± 8.21 | 74.81 ± 2.71 |


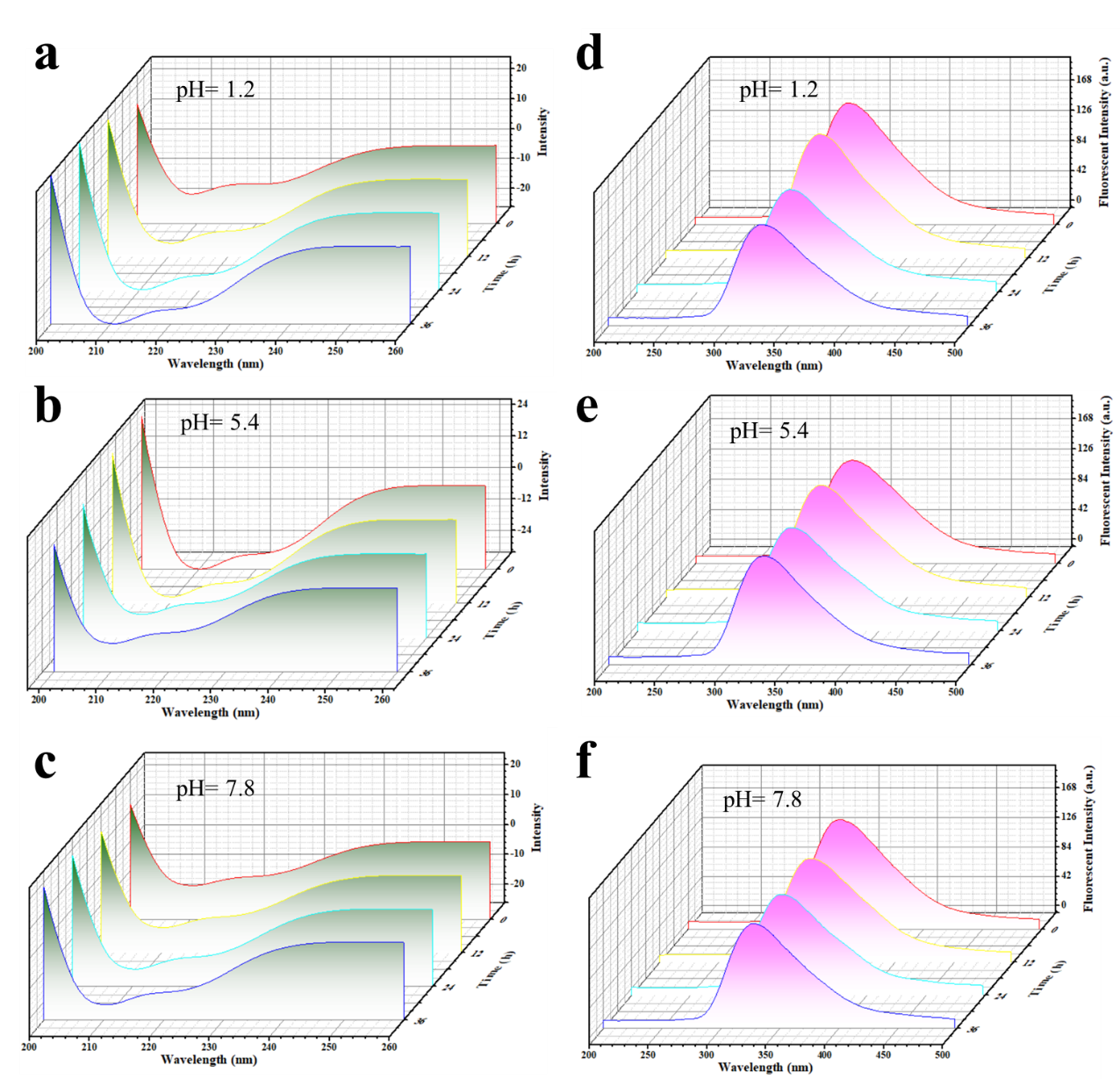


**Figure S1**. (a-c) Far−UV CD spectra of INS for period of 36h. (d-f) Fluorescence spectra of INS for period of 36h.


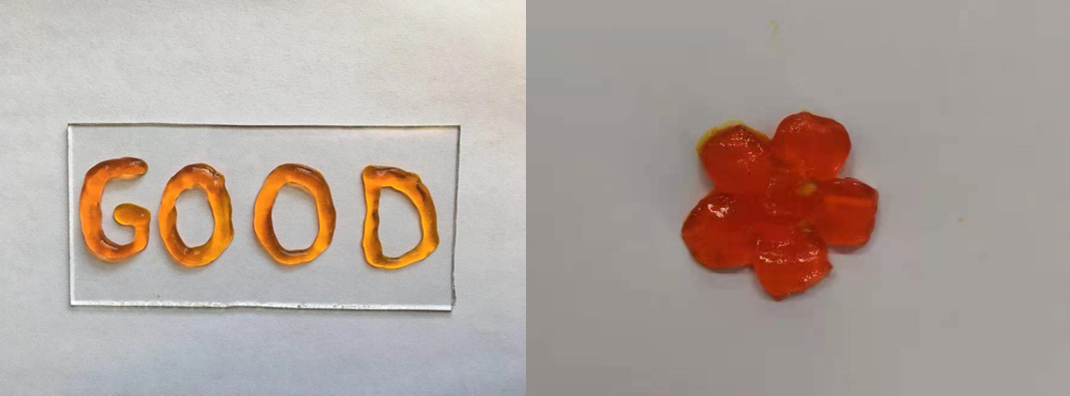


**Figure S2** Morphology of BA-Al NPs.


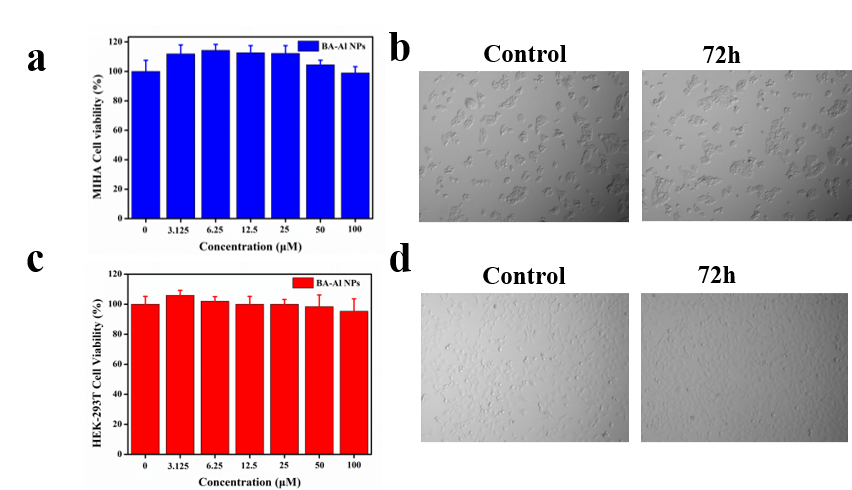


**Figure S3** Cell viability of MIHA cells incubated with BA-Al NPs for 72 h(**a**); MIHA cells image of the control group and the 72 h group treated with BA-Al NPs for 72 h(**b**); Cell viability of HEK-293T cells incubated with BA-Al NPs for 72 h(**c**); HEK-293T cells image of the control group and the 72 h group treated with BA-Al NPs for 72 h(**d**).
